# MendeLIMS: a web-based laboratory information management system for clinical genome sequencing

**DOI:** 10.1186/1471-2105-15-290

**Published:** 2014-08-27

**Authors:** Susan M Grimes, Hanlee P Ji

**Affiliations:** Stanford Genome Technology Center, Stanford University, Palo Alto, CA 94304 USA; Division of Oncology, Stanford University School of Medicine, Palo Alto, CA 94304 USA

**Keywords:** Next generation sequencing, Clinical studies, Laboratory information management, Pathology, Genomics, Genetics

## Abstract

**Background:**

Large clinical genomics studies using next generation DNA sequencing require the ability to select and track samples from a large population of patients through many experimental steps. With the number of clinical genome sequencing studies increasing, it is critical to maintain adequate laboratory information management systems to manage the thousands of patient samples that are subject to this type of genetic analysis.

**Results:**

To meet the needs of clinical population studies using genome sequencing, we developed a web-based laboratory information management system (LIMS) with a flexible configuration that is adaptable to continuously evolving experimental protocols of next generation DNA sequencing technologies. Our system is referred to as MendeLIMS, is easily implemented with open source tools and is also highly configurable and extensible. MendeLIMS has been invaluable in the management of our clinical genome sequencing studies.

**Conclusions:**

We maintain a publicly available demonstration version of the application for evaluation purposes at http://mendelims.stanford.edu. MendeLIMS is programmed in Ruby on Rails (RoR) and accesses data stored in SQL-compliant relational databases. Software is freely available for non-commercial use at http://dna-discovery.stanford.edu/software/mendelims/.

**Electronic supplementary material:**

The online version of this article (doi:10.1186/1471-2105-15-290) contains supplementary material, which is available to authorized users.

## Background

With next generation DNA sequencing (NGS) now being a commonly adopted technology, the genetic analysis of large clinical populations has become practical and is widely used for identifying disease-related germline and somatic variants such as cancer mutations. The genetic variation from thousands of individuals can now be identified with NGS whole genome, exome, targeted and other resequencing approaches. Due to the dramatic increase in the number of NGS clinical genomics studies, it has become increasingly important to develop adequate laboratory information managements systems (LIMS) to manage the thousands of patient samples that are subject to NGS analysis. Tracking and managing the clinical sample workflow involved in NGS analysis is an extremely difficult task, given the logistical issues of enrolling patients, fragmented procedures for acquisition of clinical study samples, complex molecular preparation steps and the intricacies of the NGS processing pipeline. Commercial systems are available but typically are high cost and require extensive modification to address the specific needs of biomedical research groups conducting genetic analysis on populations.

As a general and unique solution to the needs of managing the experimental workflow for clinical genome sequencing projects, we developed MendeLIMS, a web-based, robust and flexible solution for integrating the management of clinical study samples and NGS processes. With respect to genetic studies, MendeLIMS functionality can be grouped into four major categories: (i) enrollment of patients and acquisition of clinical study samples, (ii) sample assessment and processing, (iii) genomic analysis through preparation of next generation DNA sequencing libraries or other molecular assays such as microarrays and finally, (iv) DNA sequencing of samples with associated quality control metrics. Tracking of sequencing steps is currently supported for the following Illumina NGS instruments: GAIIx, MiSeq, HiSeq, HiSeq2500, NextSeq but can easily be configured for any type of NGS instrument which follows a sequencing library to flow cell workflow. We maintain a publicly available demonstration version of the application for evaluation purposes at http://mendelims.stanford.edu.

## Implementation

**Figure 1:**
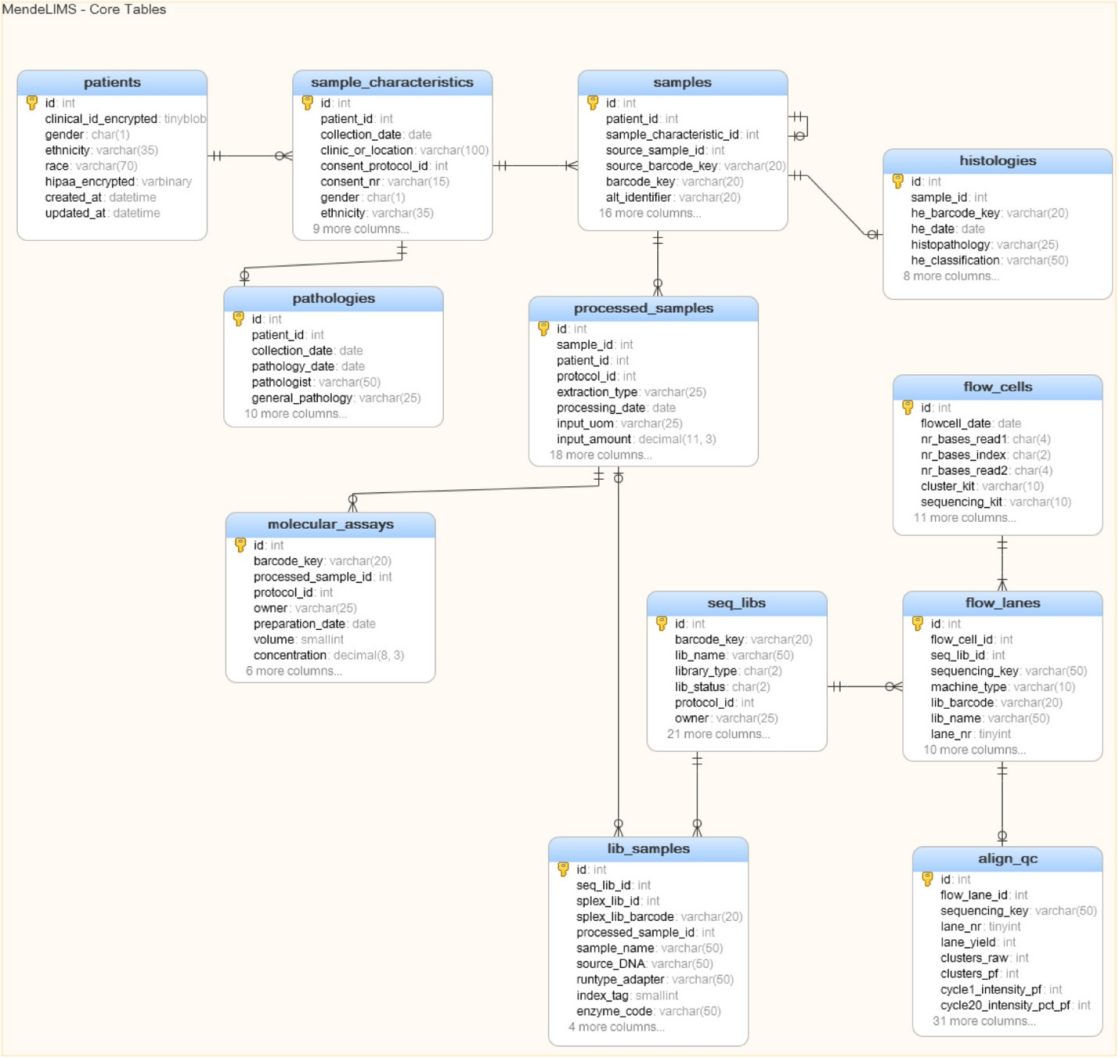
**Database schema for MendeLIMS.** Main entities and their relationships are shown in this diagram, and a complete schema showing other ancillary tables is provided in the supplementary material.

MendeLIMS is written in Ruby using the open source web application framework Ruby on Rails (RoR) and implementation is platform-independent. Instructions for installation are provided in Additional file 1. For our own in-house instance of MendeLIMS, our servers run Linux/Ubuntu and we use the MySQL relational database management system (RDBMS). The application is easily configured to use any other SQL RDBMS supported by RoR. Figure 1 shows a simplified database schema for the major tables. A more comprehensive schema is provided in Additional file 2.

**Figure 2:**
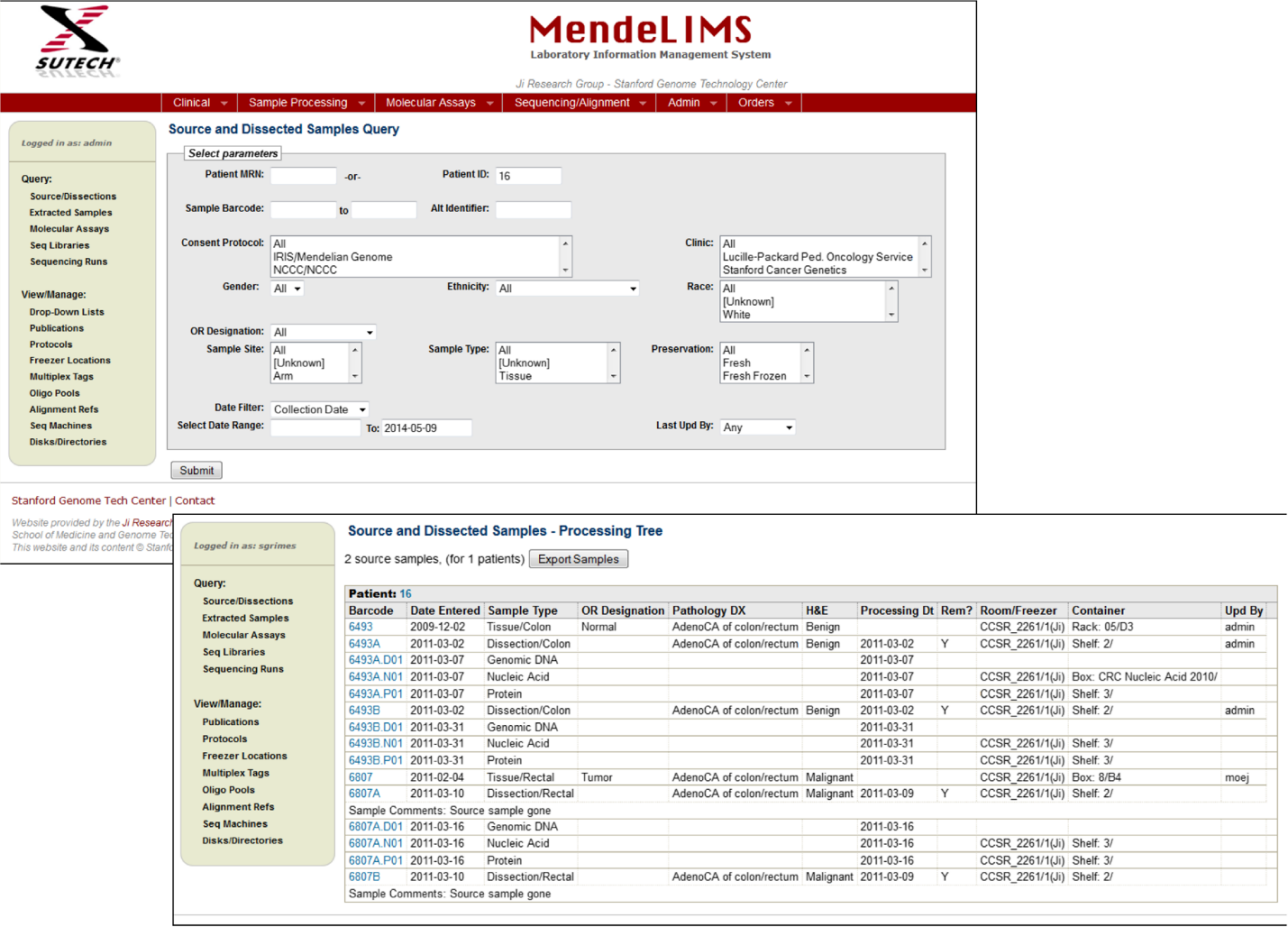
**Query web interfaces for MendeLIMS.** Database queries are managed by a series of web pages that have a modular format. Different search parameters are included with drop down menus used for standardized search terminology. Based on the needs of any given group, the search interface can be easily modified to accommodate new search or entry functions.

The web interface is designed to handle a variety of queries in a modular format (Figure 2). To facilitate consistent data entry, MendeLIMS uses drop-down lists for seamless data validation whenever possible. The drop-down lists themselves are user-configurable by users with the appropriate authorization. Examples of user-configurable items include sample types, sequencing library multiplexing schemes, alignment references and DNA sequencers. All of the features are described in the user’s manual (Additional file 3).

The look and feel of the application is easily changed or customized since all web pages inherit styles from an application-wide cascading style sheet (CSS), and in keeping with RoR convention, overall page layout and navigation is controlled by a single HTML layout file.

### Sample nomenclature

To enable accurate tracking of samples from their initial acquisition, through all key intermediate steps and ultimately to DNA sequencing, we developed a sample labeling nomenclature loosely based on the scheme employed by the Cancer Genome Atlas (TCGA) project (https://wiki.nci.nih.gov/display/TCGA/Working+with+TCGA+Data). We maintain the original unique sample barcode, and add successive suffixes to indicate processing performed.

### Acquisition of clinical study samples

**Figure 3:**
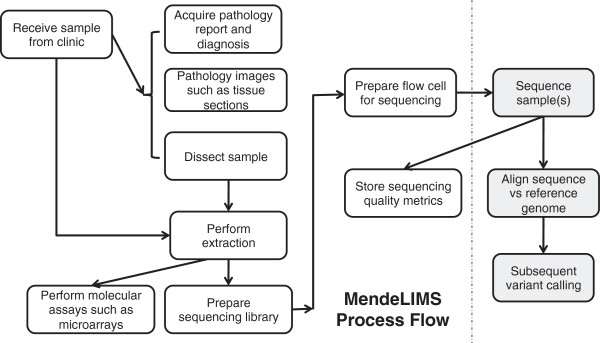
**Workflow of MendeLIMS.** Multiple steps of the sample acquisition workflow for clinical studies are fully integrated with next generation or genomic assay procedures. This allows one to trace the genomic analysis of any given sample.

After enrollment into a study, patient samples and their characteristics are entered into MendeLIMS through a web interface (Figure 2). A unique identifier (ID) is assigned for each new clinical sample. The user has the option of entering sample-relevant clinical data including pathology information from clinical reports, digital images originating from pathology slides and general clinical information about the patient (Figure 3). For efficient subsequent retrieval of the physical samples, the storage freezer and container location is entered using a standard nomenclature. If email triggers are configured, an email is automatically sent to an identified central coordinator and/or to a specified owner for the particular clinical trial giving details of any new sample entered into the system. The web interface enables sample entry to occur at any location thus facilitating sample entry by various researchers and clinical coordinators.

### Clinical study sample assessment and processing

Any manipulation of clinical study samples is tracked (Figure 3). This includes dissection of tissue samples and nucleic acid extraction. Details of these sample workflow operations are stored in MendeLIMS including volumes of any extracted macromolecules such as genomic DNA, concentration metrics and sample storage location. This greatly facilitates the managements of these precious resources for population studies.

### Tracking molecular and genomic analysis

Molecular assays and sequencing library steps are also captured in MendeLIMS (Figure 3). Sequencing libraries may be entered as singleplex (e.g. one sample per library), or multiplex (e.g. multiple samples per library with each sample tagged with a unique starting sequence). The multiplex indexing schemes are user-configurable, both for number of samples which can be multiplexed on one lane, and for the actual starting sequences used.

### Tracking the next generation sequencing workflow

**Figure 4:**
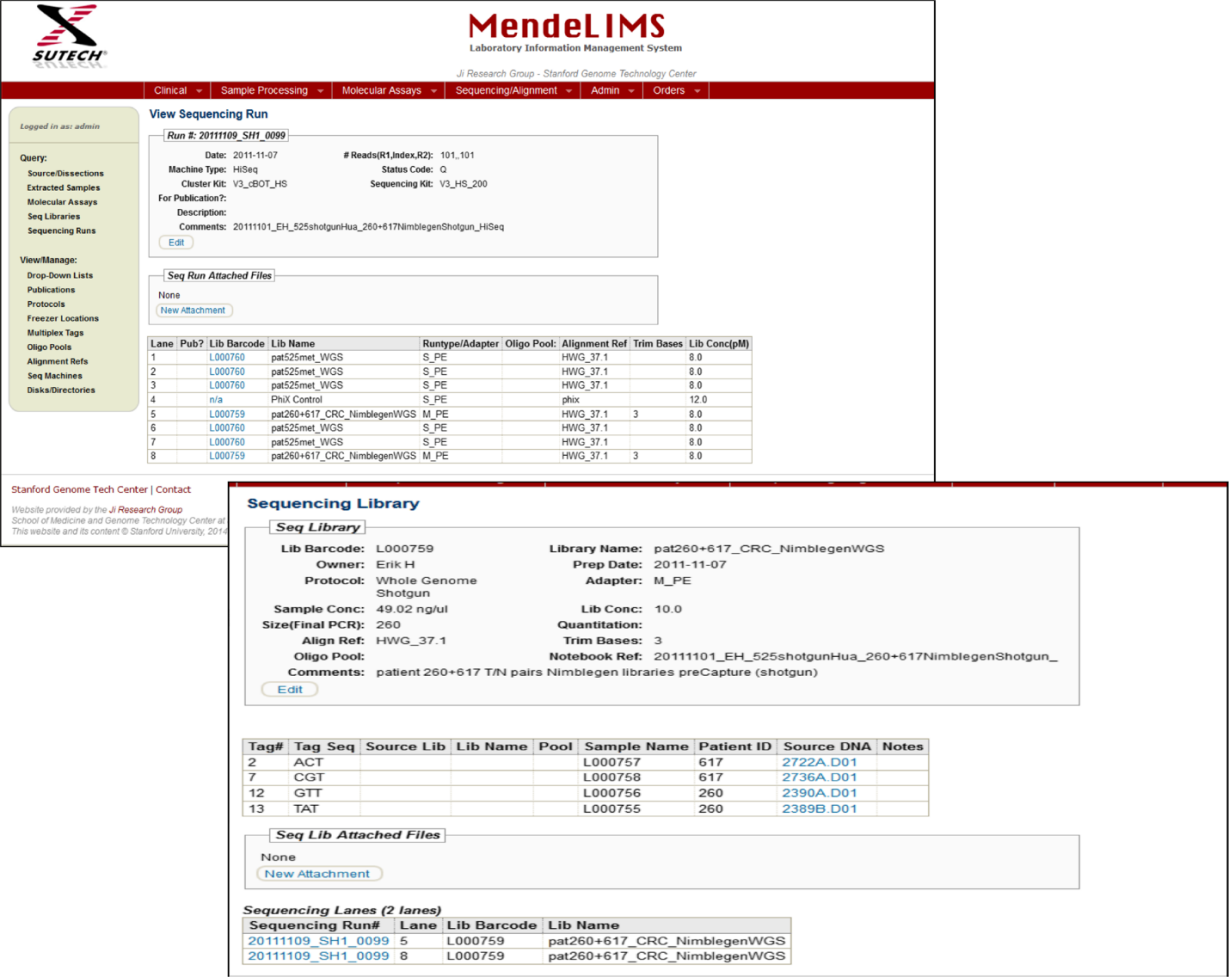
**Tracking the sequencing of clinical samples.** One can follow a clinical sample from enrollment in a clinical study all the way through to its sequencing. For example, from the sequencing run composition one can back track to the individual libraries and the original source DNA. Screen shots show the various levels of querying. A sequencing library can be queried for additional information. When required, it is possible to even determine the time of enrollment in a study and pull up relevant images from pathology.

In preparation for initiation of an NGS analysis, a sequencing run is entered into the system by selecting existing libraries and placing them into separate lanes or partitions. Normally an entire sequencing run is entered. However, the system is also able to handle partial sequencing runs to accommodate the scenario where sequencing may be performed as a service and the run is shared between multiple groups who are not privy to each other’s results. MendeLIMS generates a unique sequencing run key based on the sequencing date, sequencing machine, and a unique sequential run number. Once the sequencing run has completed, the initial quality control (QC) metrics for the run can be entered into the system. This is currently handled by an offline ruby script, but will in future be incorporated into the web application. MendeLIMS supports any type of sequencing application including whole genome, exome, targeted and RNA-based sequencing studies. The system stores sequencing library and sample lineage, flow cell composition, and sequencing run metadata, along with run status and QC metrics for all runs (Figure 4). The sequencing data files - for example bam alignment files, or vcf variant calling files - are not stored in MendeLIMS per se but are on a storage cluster accessible to all researchers in the group. Additionally, since we use the MendeLIMS sequencing run key and sequencing library/sample nomenclature in the analysis directory and file names, the files are easily cross-referenced between MendeLIMS and the storage cluster.

### Queries of MendeLIMS

All queries allow specification of multiple filter criteria such as barcode range, date range, owner, protocol which enables users to quickly find the samples of interest, and then drill-down to more detail. For example, when viewing the sample query result set (Figure 2), clicking on the sample barcode will bring up more comprehensive information regarding the sample, including pathology information. Clicking on the ‘QC’ link from a sequencing library query result set shows QC data for all sequencing runs for that library. Query results may be exported to a tab-delimited file for review or for incorporation with other local data.

### Reagent tracking

Reagents, equipment and supplies ordering, though not necessarily typical to a LIMS implementation, have been included in MendeLIMS. This feature enables for example the tracking of reagent and supply batches that is useful in troubleshooting failed sequencing runs, or the tracking of all expenditures against a specific funding account.

### Security

Hypertext Transfer Protocol Secure (HTTPS) is supported and is currently implemented for user login pages, but is easily extended to other pages as needed. User authentication is via a userid and password, and access to functionality is controlled via user roles which are defined and managed from the website by a user with ‘admin’ role. Other roles available include ‘clinical’ which allows create/modify access to clinical study sample information, ‘clin_admin’ which allows modification to drop-down lists used for system validation for sample data, ‘researcher’ which allows create/modify access to sequencing libraries and sequencing runs. The user manual in the supplementary material provides descriptions of all available user roles.

Given the extreme complexity of dealing with private health information (PHI), MendeLIMS is not designed to incorporate PHI-related clinical data. MendeLIMS does store a patient identifier that is the link between MendeLIMS and other patient clinical information databases that are securely stored in a very limited access environment. The identifier is stored as a binary encrypted field in the MySQL database and access to this field via the web application is limited to users with a ‘clinical’ or ‘clin_admin’role; other users only see a unique system-generated patient identifier which for all intents and purposes is anonymous.

In our current implementation used by several groups at Stanford University, MendeLIMS is integrated into an internal network, within a secured firewall. All database transactions are logged and time-stamped to provide an audit trail, and automated database backups are run daily. An administrator can readily generate an audit report to keep track of changes made by users.

## Discussion

There are commercial LIMS solutions available for NGS labs, some of which have been implemented at major genomic research centers. For example GeneSifter LAB Edition [1] has been implemented at Vanderbilt University; Progeny LIMS [2] at Pittsburg University and Clarity LIMS [3] at University of Washington. These systems have significant capabilities. However, the cost in time and money to implement them is often out of reach for smaller organizations, particularly those who rely on funding from research grants or who require unique workflows that can not be implemented readily in a system designed for a larger institution. Given these resource constraints, open source options are of greater interest to this category of organizations.

There are several simpler LIMS systems covering clinical study samples such as BonsaiLIMS [4], PASSIM [5] and SLIMS [6]. These LIMS offer basic sample management but do not offer comprehensive clinical sample tracking or the ability to define sequencing libraries and flow cell/sequencing run composition for NGS processing. More recent offerings which are available for open source installation and do support NGS processing include Galaxy LIMS [7] and GNomEx LIMS [8]. These systems address the flow from DNA/RNA extraction to sequencing library to flow cell/sequencing run. GNomEx LIMS also provides some analysis workflow capability and integrates data visualization via genome browsers such as UCSC Genome Browser [9] or Integrative Genomics Viewer (IGV) [10]. Galaxy LIMS takes advantage of the Galaxy infrastructure to also provide analysis workflow and data visualization. However, none of these systems natively provide tracking of clinical study data such as consent protocols, pathology and histopathology information. Sample lineage and sample tracking via consistent nomenclature, drill down to various levels of source data, and freezer container/location information is also not addressed.

Another open source option is QTREDS [11]. This LIMS has a strong focus on experimental protocols for sample preparation and tracks detailed steps which MendeLIMS and other systems do not specifically track such as sonication, end repair or ligation as part of exome library preparation. QTREDS also manages inventory of reagents for sample preparation and triggers low stock level alerts. However there is no tracking associated with clinical study samples, and NGS support is limited to the sequencing libraries and their associated sequencing status, rather than flow cell composition and sequencing run itself. In contrast, MendeLIMS provides full sample lineage tracking back to patient and the clinic and consent protocol where the sample originated, as well as support for all major processing steps through to the DNA/RNA sequencing and QC (Table 1). Additional functionality that is useful for tracking is data related to which cluster and sequencing kit versions were used for a particular run, and what publications (if any) reference the results from that run.

**Table 1:** **Comparison among different LIMS systems**

LIMS software	MendeLIMS	GNomEx	Galaxy LIMS	QTREDS
***Clinical study patient samples***				
Patient data (gender/race, MRN, pathology, histology)	Yes	No	No	No
Sample processing (dissections, extractions)	Yes	No	No	Yes^a^
Sample location tracking	Yes	No	No	Yes
***Arrays, libraries, sequencing runs***				
Molecular assays (genomic arrays, ddPCR, ..)	Yes	Yes	No	Yes
Sequencing library prep (singleplex and multiplex)	Yes	Yes	Yes	Yes^b^
Flow cell/sequencing run setup	Yes	Yes	Yes	No
***Post-sequencing analysis***				
Sequencing QC	Yes	*nd*	Yes^c^	No
Analysis workflow	No	Yes	Yes^c^	No
***Security/Audit trail***				
Authorization via user roles	Yes	Yes	Yes	Yes
Audit trail	Yes	Yes	*nd*	*nd*
HTTPS/SSL security	Yes	*nd*	*nd*	Yes
***General***				
Email/notification capability	Yes	Yes	Yes^c^	Yes
Customizable lists for data validation	Yes	Yes	Yes	Yes
Instrument integration	No	No	Yes^d^	No
Attach files to samples/libraries/sequencing runs	Yes	*nd*	No	*nd*
Visualization of results	No	Yes^e^	Yes^c^	No
***Other***				
Project based billing	No	Yes	No	No
Reagent inventory management	No	No	No	Yes
Publicly available User Guide/Demos	Yes	Yes	No	Yes

^a)^Detailed information tracked regarding sample prep; ^b)^Singleplex libraries only; ^c)^Functionality provided via integration with Galaxy and genome browsers; ^d)^Integration with HiSeq 2000 only; ^e)^Functionality provided via integration with GenoPub.

nd - Indicates that unable to determine from public documentation whether the functionality is provided.

## Conclusions

Clinical population studies using NGS sequencing require management of hundreds if not thousands of samples, including various intermediate processing steps, and the resulting sequencing data. A LIMS system is critical to the effective management of this data and the generation of reproducible results. The currently available open source or commercial systems may meet the needs of some research groups, however for those groups where the time and monetary cost of a comprehensive commercial system is prohibitive, there is no end-to-end open source solution covering enrollment of patients in a clinical study through genome sequencing analysis. Our system addresses all of these needs with a specific focus and seamless integration of clinical study enrollment through to NGS.

In MendeLIMS, all data is consolidated into one authoritative centrally accessible source repository, eliminating multiple distributed spreadsheets. Samples are traceable from a lane on a sequencing run, back to the patient diagnosis, pathology, and all processing steps in between. In conjunction with a standard barcoding nomenclature and flexible query capability, this significantly reduces errors in sample tracking, provides a comprehensive view of data being sequenced and has resulted in MendeLIMS becoming an invaluable tool for the management of our clinical sequencing studies.

## Availability and requirements

- **Project name:** MendeLIMS
- **Project home page:**http://dna-discovery.stanford.edu/software/mendelims/
- **Project demo site:**http://mendelims.stanford.edu/
- **Operating system(s):** Platform independent
- **Programming Language(s):** Ruby, Ruby on Rails, HTML, Javascript
- **Server requirements:** Apache2, Mongrel or Passenger, Ruby 1.9.3+, Rails 3.2.x, MySQL 5.0Web browser requirements: Firefox, Chrome, IE, Safari
- **License:** Any restrictions to use by non-academics: None

## Electronic supplementary material

Additional file 1: **An installation guide for MendeLIMS.** (PDF 1016 KB)

Additional file 2: **MendeLIMS database schema diagram.** (PDF 54 KB)

Additional file 2: **User’s guide for MendeLIMS.** (PDF 338 KB)

## Abbreviations

NGS: Next generation sequencing
LIMS: Laboratory information management system
RoR: Ruby on rails
RDBMS: Relational database management system
CSS: Cascading sheet style
TCGA: The cancer genome atlas
ID: Identifier
QC: Quality control
HTTPS: Hypertext transfer protocol secure
PHI: Private health information
IGV: Integrated genome viewer.
