# Supplementary material for: MendeLIMS: a web-based laboratory information management system for clinical genome sequencing
Source: BMC Bioinformatics. 2014 Aug 27;15(1):290. doi: 10.1186/1471-2105-15-290 (PMC4155081; doi:10.1186/1471-2105-15-290)
Supplement: Supplementary file 1 — Additional file 1: An installation guide for MendeLIMS. (PDF 1016 KB) [file 12859_2013_6555_MOESM1_ESM.pdf]

MendeLIMS

A web-based laboratory information management system for clinical genome sequencing

INSTALLATION GUIDE

MendeLIMS Installation Guide

Contents

Versions/Change log 3
About MendeLIMS 3
Overview 3
Accessing MendeLIMS demo site 3
MendeLIMS Installation 5
Obtaining MendeLIMS 5
Pre-requisites 5
Installation 6
Configuration Files 6
Ruby Gem Installation 7
Database Initialization 7
Starting Web Server 8
Troubleshooting 9
Moving To A Production Environment 9
Recommendations and Resources 9

MendeLIMS Installation Guide

Versions/Change log

Author: Sue Grimes

Date	Doc version	MendeLIMS version	Notes
9/15/2012	1.0	2.3	Initial documentation
6/3/2013	1.1	2.3	Reformatting, focus on development environment setup
5/8/2014	1.2	3.0	Modifications for Rails 3.0 upgrade

About MendeLIMS

Overview

MendeLIMS is a web based laboratory information management system for next generation DNA sequencing analysis of clinical populations. It provides the capability to input, annotate and query samples, sequencing libraries and sequencing runs for next generation DNA sequencing applications. It allows clinical coordinators, researchers and bench scientists to easily enter and access data from anywhere there is an internet connection. MendeLIMS is written in Ruby on Rails (http://rubyonrails.org) and data tables can be stored in any relational database management system (RDBMS) supported by Ruby on Rails, such as MySQL, PostgresSQL, SQLLite.

Accessing MendeLIMS demo site

We maintain a demonstration version of the application for evaluation purposes, which is available at: http://mendelims.stanford.edu You may sign up as a new user with your own id and password, or log in as 'admin' user with the password: 'demo'.

A suggested flow for reviewing some basic capabilities of the system:

From left navigation:
Query;Source/Dissected Samples
- leave all parameters blank, and you'll get a list of the samples in the system
- you can click around on any links to get an idea of the drill-down capability

Query;Sequencing Libraries
- Use a range of 500 to 750 for the libraries, and blank out the 'From Date'
- Again you can click around on the links

Query;Sequencing Runs
- Use a date range of 2010-10-01 to today
- Click 'Show' next to run ..SG1_0052
- Click on library: L000550

- Click on Source DNA: 00001A

This will give you an idea of the drill-down from sequencing run all the way back to the patient.

The top navigation tabs are for data entry. The process flow would typically go from left to right, starting with entry of clinical samples, then sample processing (dissection/extraction), molecular assays (eg CGH arrays) if applicable, then sequencing libraries, flow cells/sequencing. You can try this out also, and enter any test data that you wish to. Note however that test data may be re-initialized periodically which will delete any user-entered data.

MendeLIMS Installation Guide

MendeLIMS Installation

Obtaining MendeLIMS

MendeLIMS code is stored on github, and can be accessed from:

http://dna-discovery.stanford.edu/software/mendelims/

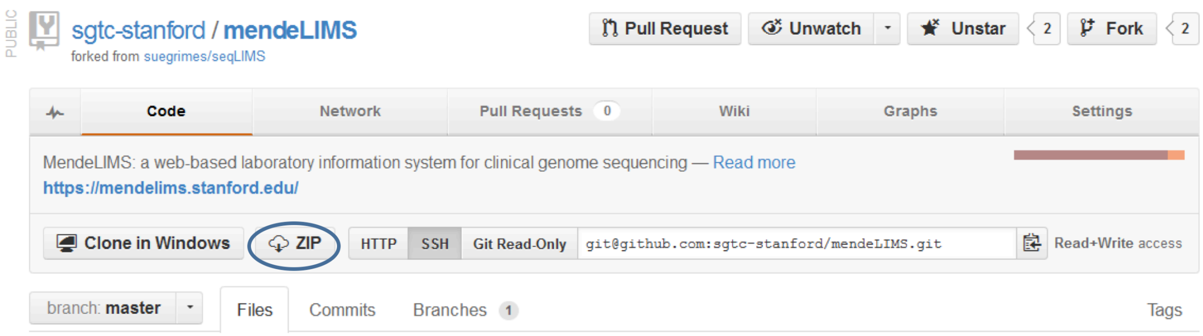

Click on the ZIP button to download a zip file of application code. Or if you have git software and are familiar with git repositories, you can clone the repository using the Git Read-Only link above: git@github.com:sgtc-stanford/mendeLIMS.git

Pre-requisites

MendeLIMS requires the following software and Ruby gem packages to be installed. Using a package manager such as apt-get or yum is recommended on Linux machines. The application currently requires rails 3.2.x and ruby 1.9.3.

Software	Version	Example install command
ruby	1.9.3	`sudo apt-get install ruby=1.9.3`
rubygems	2.2.2	`sudo apt-get install rubygems`
RDBMS: MySQL, PostgreSQL, or SQLite	Eg. MySQL, version 5.1	`sudo apt-get install mysql-server mysql-client`<br>`sudo apt-get install libmysql-ruby libmysqlclient-dev`

Ruby gems: To install ruby gems on a Linux system:

```
sudo gem install <gem_name>  -v <version>
```

Gem	Purpose	Version (latest tested version)
rails	Rails application framework	3.2.13

MendeLIMS Installation Guide

Installation

MendeLIMS is a standard Ruby on Rails application, and is relatively simple to install once pre-requisites are installed. For purposes of this document, installation to a local machine and development environment will be described. First download/extract the application files to a folder on a local hard drive, for example: /www/var/mendeLIMS. We will refer to this directory as RAILS_ROOT as per Rails convention.

Configuration Files

<RAILS_ROOT> will be used to refer to your specific RAILS_ROOT directory. Eg if you install to /www/var/mendeLIMS, <RAILS_ROOT>/config refers to /www/var/mendeLIMS/config.

Copy the following files from: <RAILS_ROOT>/config/install_examples
to: <RAILS_ROOT>/config

File	Purpose	Modifications needed
database.yml	Names and login credentials for application databases	Modify with your desired database names and login credentials. It is recommended to include at least the following database environments: development, test, production. If using the production environment, you will also need to set oligo_inventory to the same database as production.

Copy the following files from: <RAILS_ROOT>/config/install_examples
to: <RAILS_ROOT>/config/initializers

File	Purpose	Modifications needed
secret_token.rb	Secret key for verifying the integrity of signed cookies	Modify the secret token to be a string of at least 30 random characters (100 or more recommended), which will be unique to your version of the application
session_store.rb	Session key and secret token for database-stored sessions	Modify the session key and secret to be specific to your application. Secret token should be a string of at least 30 random characters (100 or more is recommended)
setup_mailer.rb	Setup for email protocol for emails which are triggered by the application	Modify to use valid smtp address , port and domain for your organization

Copy the following files from: <RAILS_ROOT>/ public/system_examples

To: <RAILS_ROOT>/ public/system

File	Purpose	Modifications needed
environment.txt	Specify root url for specific environment	Modify to specify correct url. For an initial development environment on a local machine, this will usually be http://localhost:3000
ez_keys.txt	Password and salt for ezcrypto encryption algorithm	Create and enter specific password and salt for your application.
emails.txt	Configuration for automatically triggered emails	Modify to specify whether emails are automatically triggered or not, and if so, specify correct to and from email addresses. See README_emails.txt in system_examples directory for details on specific parameters. Initially you will probably want to set this to NOEMAIL.

Note: Since some of these files contain passwords, you may wish to store the physical files elsewhere on your file system, and use symlinks to reference them at the locations above. If you are using git the files above are in .gitignore.

Ruby Gem Installation

From the command line, navigate to your RAILS_ROOT directory, and enter the following command:

```
bundle install
```

Installs all ruby gems and their associated dependencies, for gems listed in application Gemfile.

Database Initialization

Review the database.yml file which you updated in the 'Configuration' section above, and ensure that the username specified has create, insert, update, and select privileges for the database(s) specified.

From the command line, navigate to your RAILS_ROOT directory, and enter the following commands:

```
rake db:create RAILS_ENV=development
```

Creates the database specified in the 'development' block in database.yml

```
rake db:schema:load RAILS_ENV=development
```

Creates all required tables in the database specified in the 'development' block in database.yml

```
rake db:seed RAILS_ENV=development
```

Runs code in db/seeds.rb to add initial values to system tables, eg users/roles, alignment references, categories for drop-down lists

MendeLIMS Installation Guide

Starting Web Server

From the command line, navigate to your RAILS_ROOT directory, and start the built-in Rails web server by entering the following command:

```
script/server
```

From any standard web browser (Internet Explorer, Firefox, Safari, Chrome), go to http://localhost:3000 This should bring you to the MendeLIMS login page below:

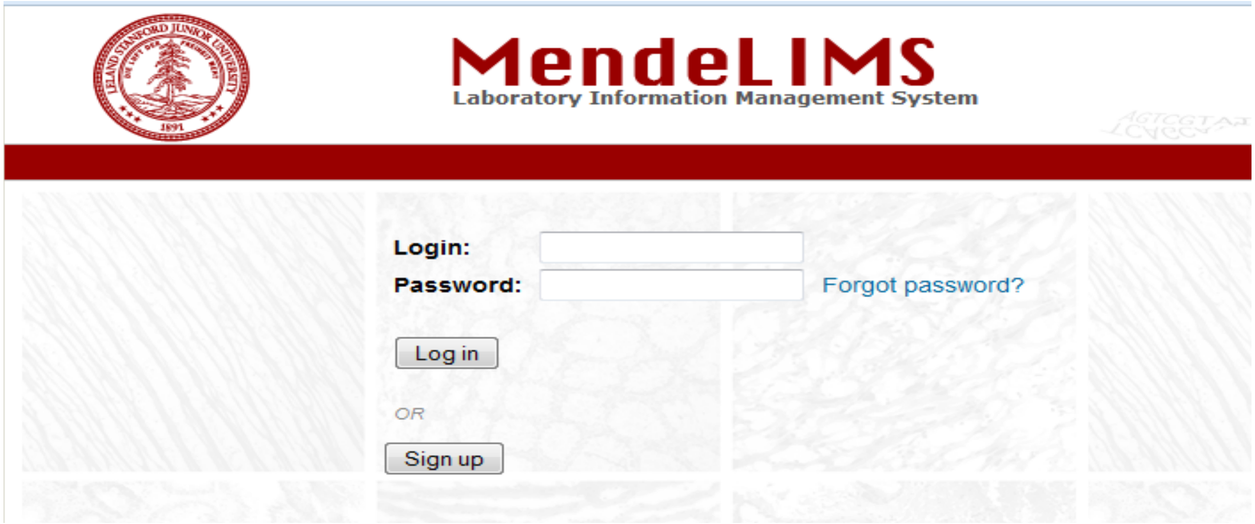

The database table initialization step will have created one user: admin, with a password: LIMS!pswd

Login with this user and password, and then for security reasons immediately change the admin email and password using the User Profile link at the top right of the top navigation bar. Note that a valid email address is critical for the ability to reset a forgotten password in the future.

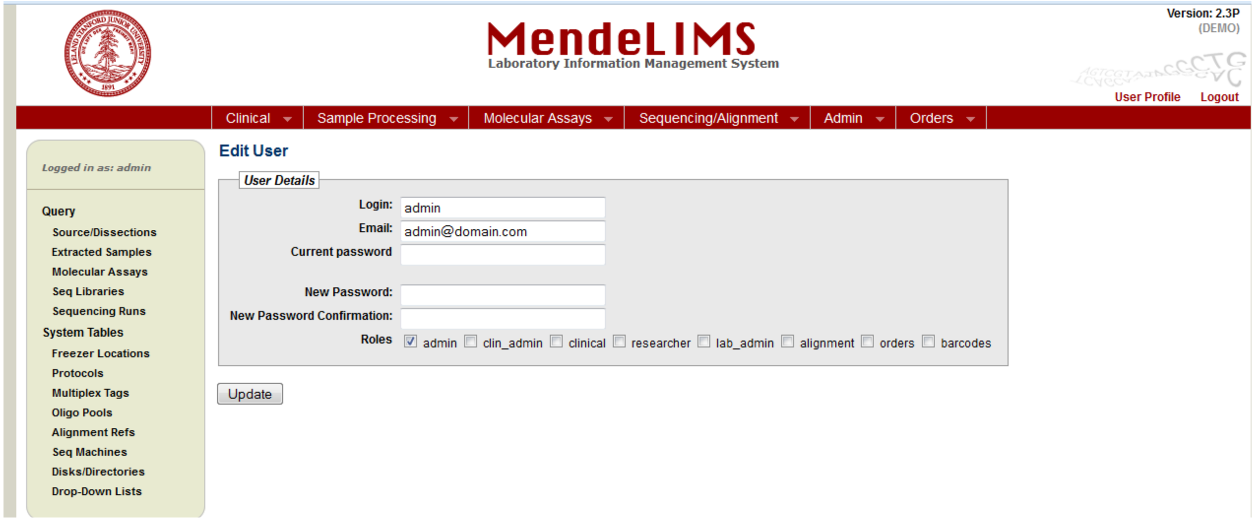

Troubleshooting

Most issues will be best handled by accessing the relevant ruby or rails community forums, such as

http://www.ruby-forum.com/forum/rails
http:// www.railsforum.com/
http://stackoverflow.com is also a good general forum for specific questions.

Moving To A Production Environment

Recommendations and Resources

To move to a production environment, an apache2/passenger web server running on an ubuntu operating system is recommended, though other options are certainly available and likely to be successful. Helpful documentation on making this transition is at: http://rubyonrails.org/deploy

Additional resources applicable to ubuntu/apache2/passenger installations and MySQL:

Apache2 web server: https://help.ubuntu.com/8.04/serverguide/C/httpd.html
Phusion Passenger application server: https://www.phusionpassenger.com/support
General Ruby on Rails: https://help.ubuntu.com/community/RubyOnRails
MySQL: https://help.ubuntu.com/8.04/serverguide/C/mysql.html
