# Supplementary material for: MendeLIMS: a web-based laboratory information management system for clinical genome sequencing
Source: BMC Bioinformatics. 2014 Aug 27;15(1):290. doi: 10.1186/1471-2105-15-290 (PMC4155081; doi:10.1186/1471-2105-15-290)
Supplement: Supplementary file 2 — Additional file 2: MendeLIMS database schema diagram. (PDF 54 KB) [file 12859_2013_6555_MOESM2_ESM.pdf]

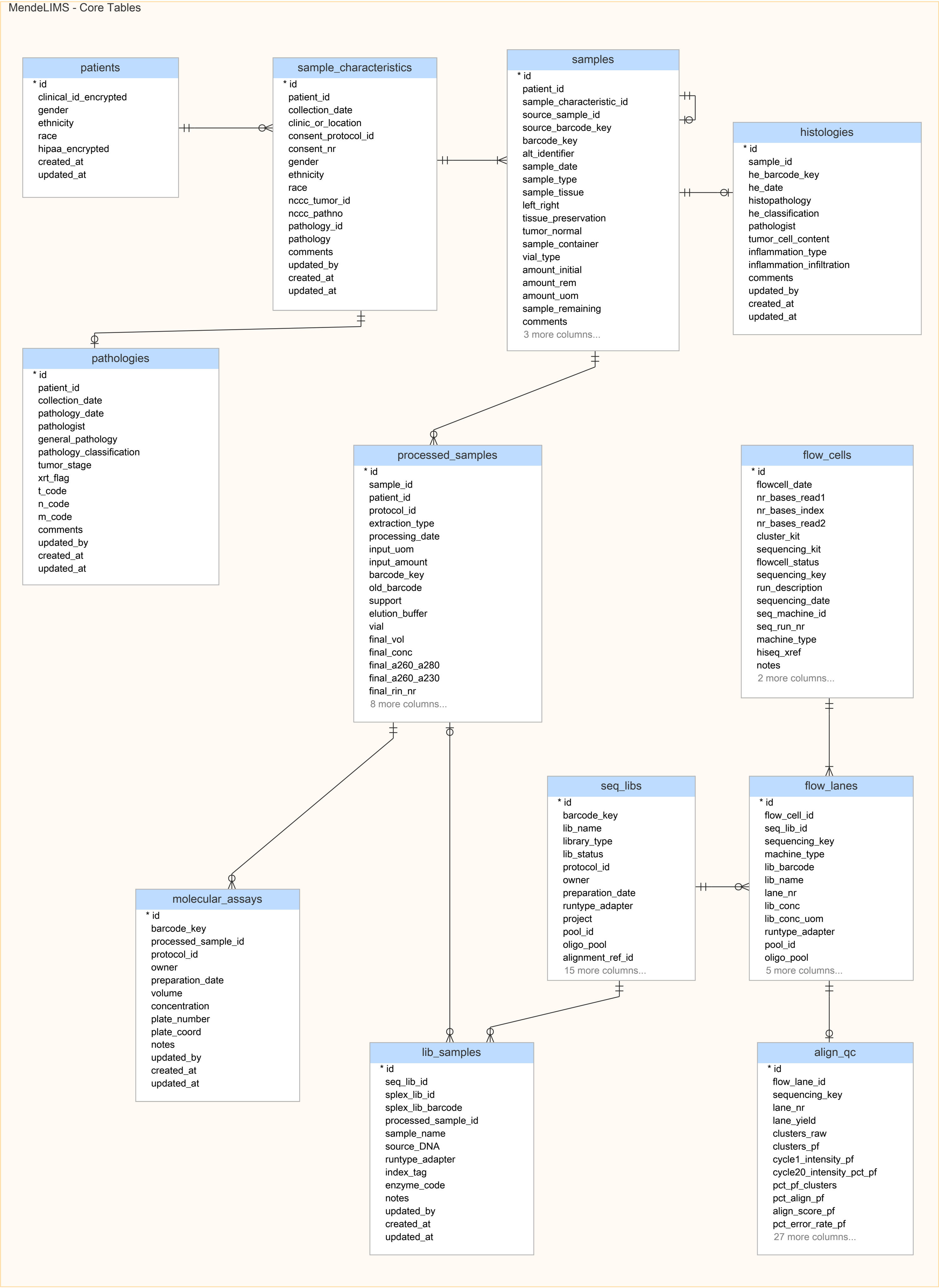
MendeLIMS - Core Tables
patients
* id
clinical_id_encrypted
gender
ethnicity
race
hipaa_encrypted
created_at
updated_at
sample_characteristics
* id
patient_id
collection_date
clinic_or_location
consent_protocol_id
consent_nr
gender
ethnicity
race
nccc_tumor_id
nccc_pathno
pathology_id
pathology
comments
updated_by
created_at
updated_at
samples
* id
patient_id
sample_characteristic_id
source_sample_id
source_barcode_key
barcode_key
alt_identifier
sample_date
sample_type
sample_tissue
left_right
tissue_preservation
tumor_normal
sample_container
vial_type
amount_initial
amount_rem
amount_uom
sample_remaining
comments
3 more columns...
histologies
* id
sample_id
he_barcode_key
he_date
histopathology
he_classification
pathologist
tumor_cell_content
inflammation_type
inflammation_infiltration
comments
updated_by
created_at
updated_at
pathologies
* id
patient_id
collection_date
pathology_date
pathologist
general_pathology
pathology_classification
tumor_stage
xrt_flag
t_code
n_code
m_code
comments
updated_by
created_at
updated_at
processed_samples
* id
sample_id
patient_id
protocol_id
extraction_type
processing_date
input_uom
input_amount
barcode_key
old_barcode
support
elution_buffer
vial
final_vol
final_conc
final_a260_a280
final_a260_a230
final_rin_nr
8 more columns...
flow_cells
* id
flowcell_date
nr_bases_read1
nr_bases_index
nr_bases_read2
cluster_kit
sequencing_kit
flowcell_status
sequencing_key
run_description
sequencing_date
seq_machine_id
seq_run_nr
machine_type
hiseq_xref
notes
2 more columns...
seq_libs
* id
barcode_key
lib_name
library_type
lib_status
protocol_id
owner
preparation_date
runtype_adapter
project
pool_id
oligo_pool
alignment_ref_id
15 more columns...
flow_lanes
* id
flow_cell_id
seq_lib_id
sequencing_key
machine_type
lib_barcode
lib_name
lane_nr
lib_conc
lib_conc_uom
runtype_adapter
pool_id
oligo_pool
5 more columns...
molecular_assays
* id
barcode_key
processed_sample_id
protocol_id
owner
preparation_date
volume
concentration
plate_number
plate_coord
notes
updated_by
created_at
updated_at
lib_samples
* id
seq_lib_id
splex_lib_id
splex_lib_barcode
processed_sample_id
sample_name
source_DNA
runtype_adapter
index_tag
enzyme_code
notes
updated_by
created_at
updated_at
align_qc
* id
flow_lane_id
sequencing_key
lane_nr
lane_yield
clusters_raw
clusters_pf
cycle1_intensity_pf
cycle20_intensity_pct_pf
pct_pf_clusters
pct_align_pf
align_score_pf
pct_error_rate_pf
27 more columns...

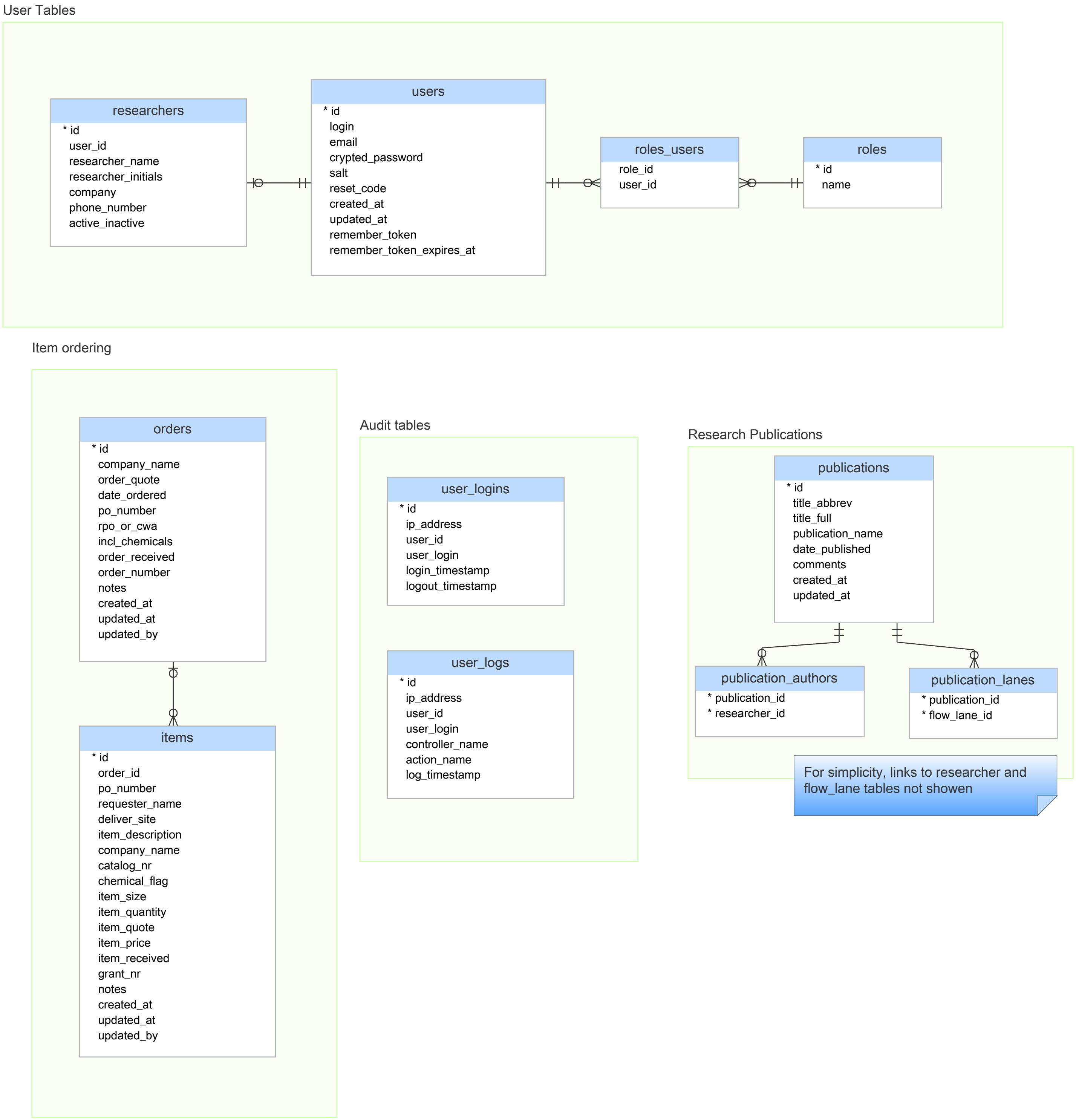

User Tables
researchers
* id
user_id
researcher_name
researcher_initials
company
phone_number
active_inactive
users
* id
login
email
crypted_password
salt
reset_code
created_at
updated_at
remember_token
remember_token_expires_at
roles_users
role_id
user_id
roles
* id
name
Item ordering
orders
* id
company_name
order_quote
date_ordered
po_number
rpo_or_cwa
incl_chemicals
order_received
order_number
notes
created_at
updated_at
updated_by
items
* id
order_id
po_number
requester_name
deliver_site
item_description
company_name
catalog_nr
chemical_flag
item_size
item_quantity
item_quote
item_price
item_received
grant_nr
notes
created_at
updated_at
updated_by
Audit tables
user_logins
* id
ip_address
user_id
user_login
login_timestamp
logout_timestamp
user_logs
* id
ip_address
user_id
user_login
controller_name
action_name
log_timestamp
Research Publications
publications
* id
title_abbrev
title_full
publication_name
date_published
comments
created_at
updated_at
publication_authors
* publication_id
* researcher_id
publication_lanes
* publication_id
* flow_lane_id
For simplicity, links to researcher and flow_lane tables not showen

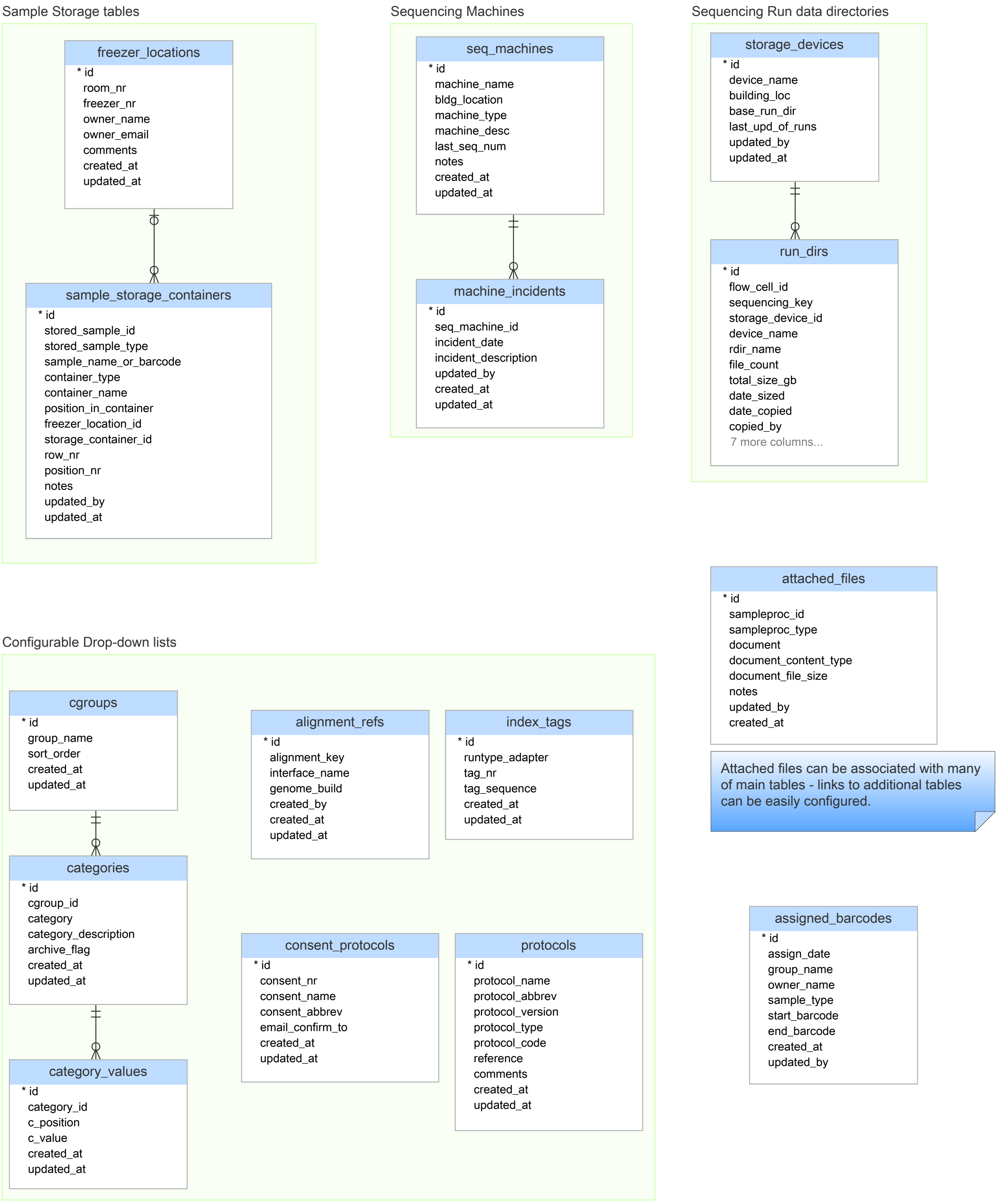

Sample Storage tables
freezer_locations
* id
room_nr
freezer_nr
owner_name
owner_email
comments
created_at
updated_at
sample_storage_containers
* id
stored_sample_id
stored_sample_type
sample_name_or_barcode
container_type
container_name
position_in_container
freezer_location_id
storage_container_id
row_nr
position_nr
notes
updated_by
updated_at
Sequencing Machines
seq_machines
* id
machine_name
bldg_location
machine_type
machine_desc
last_seq_num
notes
created_at
updated_at
machine_incidents
* id
seq_machine_id
incident_date
incident_description
updated_by
created_at
updated_at
Sequencing Run data directories
storage_devices
* id
device_name
building_loc
base_run_dir
last_upd_of_runs
updated_by
updated_at
run_dirs
* id
flow_cell_id
sequencing_key
storage_device_id
device_name
rdir_name
file_count
total_size_gb
date_sized
date_copied
copied_by
7 more columns...
attached_files
* id
sampleproc_id
sampleproc_type
document
document_content_type
document_file_size
notes
updated_by
created_at
Attached files can be associated with many of main tables - links to additional tables can be easily configured.
Configurable Drop-down lists
cgroups
* id
group_name
sort_order
created_at
updated_at
categories
* id
cgroup_id
category
category_description
archive_flag
created_at
updated_at
category_values
* id
category_id
c_position
c_value
created_at
updated_at
alignment_refs
* id
alignment_key
interface_name
genome_build
created_by
created_at
updated_at
index_tags
* id
runtype_adapter
tag_nr
tag_sequence
created_at
updated_at
consent_protocols
* id
consent_nr
consent_name
consent_abbrev
email_confirm_to
created_at
updated_at
protocols
* id
protocol_name
protocol_abbrev
protocol_version
protocol_type
protocol_code
reference
comments
created_at
updated_at
assigned_barcodes
* id
assign_date
group_name
owner_name
sample_type
start_barcode
end_barcode
created_at
updated_by
