# Supplementary material for: MendeLIMS: a web-based laboratory information management system for clinical genome sequencing
Source: BMC Bioinformatics. 2014 Aug 27;15(1):290. doi: 10.1186/1471-2105-15-290 (PMC4155081; doi:10.1186/1471-2105-15-290)
Supplement: Supplementary file 3 — Additional file 2: User’s guide for MendeLIMS. (PDF 338 KB) [file 12859_2013_6555_MOESM3_ESM.pdf]

MendeLIMS

A web-based laboratory information management system for clinical genome sequencing

USER GUIDE

MendeLIMS User Guide

Contents

Versions/Change log 4
User Access 4
  Signup, Login 4
  Change or reset password 4
Data Entry 4
  Clinical Samples 4
  Pathology Report 6
  H&E Slides 6
  Dissections 7
  Extractions 8
  Molecular Assays 8
  Sequencing Libraries 9
  Flow Cells 10
  Sequencing Runs 12
Queries 12
  General Instructions 12
  Source/Dissections 13
  Extracted Samples 13
  Molecular Assays 13
  Seq Libraries 13
  Sequencing Runs 13
Orders 13
  New Order Item 13
  Place Order for Entered Items 14
  View Order Items 15
  View Orders 15
Configurable tables 15
Admin (Users, Roles) 15
  Managing users/roles 16

Managing researchers 16

MendeLIMS User Guide

Versions/Change log

Date	Documentation version	LIMS version	Notes
11/24/2010	1.0	1.4	Initial documentation
5/24/2011	1.1	2.04	Revised for new UI, and for Molecular Assay functionality
6/3/2013	1.2	2.3	Formatting revisions, update access info
5/8/2014	1.3	3.2	Review for changes applicable to Rails 3.2/none needed

User Access

Signup, Login

If you are accessing MendeLIMS for the first time, click the 'Signup' button on the home page, to create an id and password. You will initially have access to limited functionality, and will need to contact the system administrator to have privileges established based on your research or lab role.

Change or reset password

You can update your email address or change your password after logging in, by accessing 'User Profile' at the top right above the top navigation bar. If you forget your password, you can reset it by clicking the 'Forgot Password?' link on the home page, and entering the email address that is associated with your login. You will receive an email containing a link to reset your password.

Data Entry

Clinical Samples

1. Select Samples;New Clinical Sample from top navigation bar
   System will return data entry field for Medical Record Number (MRN)

2. Enter MRN and click 'Submit'
   System will return patient id, gender, race, ethnicity if patient is already in the system, otherwise these fields will be blank/default values to allow for data entry. System will also return data entry forms for sample details.

3. Enter sample information and click 'Save'
   System will return a confirmation screen with information just added.

4. If you have multiple samples with the same characteristics (eg. several vials of blood, or a tumor/normal tissue pair), click on the line which reads:

Add another sample for this patient/collection date
System will return display an additional set of form fields for the next physical sample, which you can enter as in step 3.

Required fields:

- MRN, consent protocol, clinic, sample barcode

If drop-down values not populated with value needed:

- See 'DropDowns' section.

To correct a mistake after source sample is entered:

- Wrong MRN (new patient, MRN did not previously exist in system)
  - Go to Clinical; Edit; Patient on the top navigation bar
  - Enter mrn, patient id, or a sample barcode to retrieve patient details
  - Edit mrn# and click 'update'
- Wrong MRN (and wrong MRN incorrectly refers to an existing patient)
  - If you enter a sample with an incorrect mrn, and the incorrect mrn is an existing patient, the sample will be incorrectly attached to that patient, and will need to deleted and then re-added.
  - Go to Query; Source/Dissections on the left navigation bar
  - Provide filter parameters which will select the sample that you need to delete
  - Resulting table will have a 'delete' link on the far right of each row which will be active if the sample has had no further processing done on it, and if your login id has an 'admin' role
  - Delete the sample which is associated with an incorrect mrn#
  - Re-enter with the correct MRN
- Wrong barcode
  - Follow same procedure as wrong MRN where MRN is an existing patient
- Wrong protocol, clinic or collection date (fields in sample acquisition section of data entry form)
  - Go to Clinical; Edit; Sample Acquisition on top navigation bar
  - Enter MRN, patient id or barcode to identify sample characteristics to be updated
  - Correct erroneous fields and click 'update'
- Wrong container, amount, physical storage location etc (fields in physical sample sections of data entry form)
  - Go to Clinical; Edit; Physical Sample
  - Enter barcode to identify sample to be updated
  - Correct erroneous fields and click 'update'

Pathology Report

Pathology report is associated with a sample 'acquisition' which will roughly equate to a specific operative date, and will be associated with one patient, but may be associated with more than one physical sample. Eg tumor/normal tissue samples, or multiple vials of blood.

1. Select Clinical; New Pathology Rpt from top navigation bar
   System will request MRN or patient ID

2. Enter MRN or patient ID and click 'Submit'.
   All sample 'acquisitions' for the patient will be displayed, with those already having an associated pathology report displayed first, with the pathology details. If there are any 'acquisitions' which do not have an associated pathology report there will be an 'Add New' button under at Diagnosis/Pathology heading

3. Click 'Add New' to add a new pathology report, or 'Edit' to edit an existing pathology report. If new pathology details are entered one or more check boxes next to 'associated samples acquired' must be checked, and pathology will be associated with those records.

Required fields:

- Barcode (system generated)

If drop-down values not populated with values needed:

- See 'DropDowns' section.

To correct a mistake after pathology report is entered:

- Select Clinical; Edit; Pathology Rpt from the top navigation bar
  - Enter patient MRN or ID
  - Click 'Edit' next to pathology report to be modified
  - Correct erroneous data and click 'Update'

H&E Slides

1. Select Clinical; New H&E Slide from top navigation bar
   System will request source barcode

2. Enter the barcode for the source sample from which the H&E slide was taken, and click 'Submit'

System will return sample information, and a data entry form for H&E slide information. H&E slide barcode will be system generated as source barcode with .H01 suffix. Note that presently only one H&E slide can be entered per source sample, therefore if there is an existing H&E slide for this sample, you will be directed to an edit page.

3. Enter H&E slide details, then click 'Save'

Required fields:

- Barcode (system generated)

If drop-down values not populated with values needed:

- See 'DropDowns' section.

To correct a mistake after H&E slide is entered:

- Select Clinical; Edit; H&E Slide from the top navigation bar
    - Enter the barcode for the source sample from which the H&E slide was taken and click 'Submit'
    - Correct erroneous data and click 'update'

Dissections

1. Select Sample Processing; New Dissection from top navigation bar
   System will return data entry field for sample barcode

2. Enter the barcode for the source sample from which the dissection was taken
   System will return brief details pertaining to the source sample, and a data entry form for dissected sample details. The system will generate a unique barcode which will be the source sample barcode with a one character alpha suffix.

3. If source sample is depleted with this dissection, enter 'N' in the drop-down box in the source sample section. Enter dissection details, then click 'Save'

Required fields:

- Barcode (system generated)

If drop-down values not populated with values needed:

- See 'DropDowns' section.

To correct a mistake after dissected sample is entered:

- Select Sample Processing; Edit Sample from the top navigation bar
  - Enter dissected sample barcode
  - Correct erroneous data and click 'update'

Extractions

1. Select Sample Processing; New Extraction from top navigation bar
   System will return data entry field for sample barcode

2. Enter the barcode for the actual sample from which the extraction was taken (this will be the original source sample for blood, or typically a dissected sample for tissue).
   System will return brief details pertaining to the source sample, and a data entry form for extraction details. The system will generate a unique barcode which will be the source sample barcode with an appropriate suffix. First two characters of suffix will be '.D' for DNA, '.R' for RNA, '.N' for nucleic acid or '.P' for protein. Subsequent characters will be a 2 digit number to form a unique barcode.

3. If source sample is depleted with this extraction, enter 'N' in the drop-down box in the source sample section. Enter extraction details, then click 'Save'

Required fields:

- Barcode (system generated)

If drop-down values not populated with values needed:

- See 'DropDowns' section.

To correct a mistake after extracted sample is entered:

- Select Sample Processing; Edit Sample from the left navigation bar
  - Enter extracted sample barcode
  - Correct erroneous data and click 'update'

Molecular Assays

1. Select Molecular Assays; New Assay from top navigation bar
   System will return data entry form for molecular assay defaults

2. Enter values to be used as defaults for all assays created from this web page if applicable; Enter # assays(n)to be created with these defaults; Click 'Add samples'
System will return n data entry lines, each populated with the assay defaults

3. For each assay, enter source DNA/RNA barcode, and assay volume and concentration. Note that the source DNA/RNA barcode must already exist in LIMS. Click 'Save assays'
System will return a confirmation screen with details of the assays added, including the volume of sample to pipette from the source extraction, and the buffer volume needed. Calculations are based on the source DNA/RNA volume and concentration in the system. The system will generate a unique assay code which will be the source sample barcode with an appropriate suffix. First three characters of suffix will indicate the type of assay (eg CGH for array CGH), and subsequent characters will be a 2 digit number to form a unique assay code.

Required fields:

- Source DNA/RNA, assay volume, assay concentration

If drop-down values not populated with values needed:

- See 'DropDowns' section.

To correct a mistake after extracted sample is entered:

- Select Molecular Assays; List Assays from the top navigation bar
    - Enter extracted sample barcode
    - Correct erroneous data and click 'update'

Sequencing Libraries

Singleplex:

1. Select Sequencing/Alignment; Library Prep; New Lib-Singleplex from top navigation bar
System will return a data entry form for entering library defaults. If you will only be entering one library with similar information you may enter pertinent data on the defaults form, or just go directly to the library entry page by entering '1' as number of libraries.

2. Enter values to be used as defaults for all libraries created from this web page if applicable; Enter # libraries(n)to be created with these defaults; Click 'Add libraries'
System will return n data entry lines, each populated with the library defaults

3. For each library, enter sample name, and update any of the default values as needed. Click 'Save'.
   Note that per the nomenclature we have adopted, the library barcode must start with 'L' and be followed by 6 digits.

Multiplex:

1. Select Sequencing/Alignment; Library Prep; New Lib-Multiplex from top navigation bar
   System will return a data entry form for entering library details.

2. Enter values as appropriate. Note that the selection of adapter will determine how many samples can be multiplexed and the sequence of the index tags; Click 'Enter sample details'
   System will return the appropriate number of lines for sample entry, according to how many samples can be multiplexed given the specified adapter.

3. Enter data for each sample in the multiplex library (leaving blank the lines for any index tags which are not used). Click 'Save Library'.
   Note that if you wish to correct one of the library detail values and repopulate all sample lines, you can click 'Refresh from Defaults'

Required fields:

- Barcode, library name, owner, adapter, alignment reference

If drop-down values not populated with values needed:

- See 'DropDowns' section.

To correct a mistake after sequencing library is entered:

- Select Query; Seq Libraries from the left navigation bar
  - Provide filter parameters which will select the library that you need to edit
  - Resulting table will have an 'edit' link on the far right of each row, if you have authorization to edit libraries
  - Click 'Edit' next to the library to be modified (right most column in the list)
  - Make changes as needed and click 'update'

Flow Cells

1. Select Sequencing/Alignment; Sequencing;Prepare Flow Cell from top navigation bar
   System will return a parameter selection form to filter the libraries to be returned, and subsequently selected from, for placement on a flow lane.

2. Enter parameters as appropriate. Uncheck the 'Exclude used libraries' box if you will be selecting any libraries which have previously been sequenced.
System will return a data entry form for the flow cell details, and a list of sequencing libraries matching the filter parameters. Each sequencing library will have two data entry fields for lane number, and library concentration.

3. Complete flow cell details, and enter lane number(s) and concentration to the left of each library which is on the flow cell. Click 'create'.
Note that exactly 8 lanes per flow cell is required, unless the 'Partial FlowCell' field is marked as 'Y'. Partial flow cells must have 8 or fewer lanes which need not be sequential.

4. If the flow cell is being immediately sequenced, click 'Submit for Sequencing'.
System will return a data entry form with sequencing date (defaults to current date), and a drop down list of available sequencing machines.

5. Enter values as appropriate, and click 'Submit'.
System will generate a unique sequencing identifier which consists of the sequencing date, sequencing machine, and the next sequential run number.

Required fields:

- Number of bases (read 1), Lane number (must be numeric between 1 and 8)

If drop-down values not populated with values needed:

- See 'DropDowns' section.

To correct a mistake after flow cell is entered:

- If flow cell has not yet been submitted for sequencing, select Sequencing/Alignment; Sequencing; Sequence Flow Cell from the top navigation bar
  - Click 'Edit' to the right of the flow cell to be modified
  - Correct erroneous data and click 'update'

- If flow cell has already been submitted for sequencing, select Query; Sequencing Runs from left navigation bar
  - Click 'Edit' to the right of the flow cell/run to be modified
  - Correct erroneous data and click 'update'

MendeLIMS User Guide

Sequencing Runs

1. Select Sequencing/Alignment; Sequencing; Flow Cells for Seq from top navigation bar
   System will return a list of flow cells that have been created, but not yet sequenced.

2. Click 'Show/Seq' to the right of the flow cell to be sequenced.
   System will show the flow cell lanes/samples.

3. Click 'Submit for Sequencing'.
   System will return a data entry form with sequencing date (defaults to current date), and a drop down list of available sequencing machines.

4. Enter values as appropriate, and click 'Submit'.
   System will generate a unique sequencing identifier which consists of the sequencing date, sequencing machine, and the next sequential run number.

Required fields:

- Sequencing date, sequencing machine

If drop-down values not populated with values needed:

- See 'DropDowns' section.

To correct a mistake after sequencing run is entered:

- Select Query; Sequencing Runs from the left navigation bar
  - Click 'Edit' to the right of the sequencing run to be modified
  - Correct erroneous data and click 'update'

  Note that sequencing lane numbers can be switched if incorrect, but sequencing libraries on the flow cell cannot be changed once entered.

QUERIES

General Instructions

- Select Query;<Query subtype> from left navigation bar
  System will return a form for entering query filter parameters. Select values as appropriate to limit result set to data of interest.

Source/Dissections

- Query results will be in a tree format, showing source sample then any dissection, then any extractions from that dissection, then next dissection, etc.

Extracted Samples

- Query results will be a list of extracted samples.

Molecular Assays

- Query results will be a list of molecular assays.

Seq Libraries

- Library name parameter is a search string, so any library names containing that string will be returned. Query results will show libraries and the number of lanes on which that library has been sequenced (if any). If library has been sequenced there will also be a link to the sequencing QC results.

Sequencing Runs

- Query results will be a list of sequencing runs. Status code field will be one of the following: R (Ready for sequencing), S (picked up for Sequencing), Q (QC results loaded). If QC results have been loaded there will be a link to access those results.

ORDERS

New Order Item

1. Select Orders;New Item from top navigation bar
   System will return a data entry form for specification of general information for the order item(s), ie. Requestor, Delivery Site and Grant number.

2. Enter values as appropriate, then specify how many distinct items (n) to be ordered
   System will return a data entry form with n lines for items.

3. Enter item information, then click 'Save Items'
   Note that the following fields are auto-complete fields and will bring up a list of any existing values in the database, once 3 characters have been entered: Catalog#, Description, Company name. Additionally, if a catalog# or description is selected from the auto-complete list, item data will be auto-populated with values from the previous order – except that item quantity will be left blank for manual entry.
   Note also that any lines with a blank catalog number and blank description will be ignored.

Required fields:

- Requestor, Delivery Site, Company name, Chemical flag, Catalog #, Item Description, Item Quantity

If drop-down values not populated with values needed:

- See 'DropDowns' section.

To correct a mistake after item is entered:

- Select Orders; View/Order Items from the top navigation bar
    - Enter parameters as appropriate
    - Click 'Edit' next to item to be modified

Place Order for Entered Items

Workflow is set up for centralized ordering, so multiple researchers may enter items to be ordered, and the actual ordering happens once or twice/day by an administrator. In the demo system, the admin user has authorization to place orders.

1. Select Orders; View/Order Items from top navigation bar
   System will return a data entry form for specifying which order items to list.

2. Typically the admin would specify Status='Not Ordered', and specify the delivery site. Other parameters can be specified as needed.
   System will return a list of items satisfying the above criteria. All items which have not been ordered will have a check box to the left of the item information.

3. Check the box next to all items to be ordered on a specific PO or CWA(Corporate Wide Agreement), then click 'Place order for checked items'
   System will return a data entry form for specifying order details.

4. Enter order details and click 'Submit'

Required fields:

- RPO/CWA, Company Name, PO Number

If drop-down values not populated with values needed:

- See 'DropDowns' section.

To correct a mistake after order is entered:

- Select Orders; List/Upd Orders from the top navigation bar

  - Enter parameters as appropriate
  - Click 'Edit' next to order to be modified

View Order Items

- Select Orders; View/Order Items from the top navigation bar
  - Select values as appropriate to limit result set to data of interest.
    Note: for users with an admin role, the resulting page will also allow order to be placed for any unordered items in the list.

View Orders

- Select Orders; List/Upd Orders from the top navigation bar
  - Select values as appropriate to limit result set to data of interest.

CONFIGURABLE TABLES

Configurable tables are accessed via the System Tables menu item on the left navigation bar. Most drop-down lists on data entry screens are dynamically populated by values in one of these tables. All modification (create/update/delete) is accessed via one of the sub-menu items below:

- Freezer Locations – Physical freezer locations where samples may be stored
- Protocols – Consent protocols for clinical samples, or Sample processing protocols (eg. Extraction or Library Preparation)
- Multiplex Tags – Tag index values and tag sequence for multiplexed libraries
- Oligo Pools - If pools of oligos are used for example for targeted capture, these oligos can be stored here and referenced by sequencing library or sequencing run.
- Alignment Refs - Code which will determine which reference is used to align base calls from sequencing run.
- Seq Machines - List of sequencing machines and types (creates drop-down list used when for sequencing run is created)
- Disks/Directories – List of possible disk/directory paths, under which sequencing runs may be stored.
- Drop Downs – General drop-down lists, sorted into categories by which authorization to modify the values is assigned. Eg. A user with 'clin_admin' role can modify drop-down list values for samples, but not for sequencing libraries. Conversely a user with 'lab_admin' role can modify drop-down list values for sequencing libraries but not for source samples.

ADMIN (USERS, ROLES)

Users with an 'admin' role have the ability to assign roles to other users, and to add researchers. Only admins will see the Admin option on the left navigation bar.

Managing users/roles

- Select Admin; Users from top navigation bar
  List of system users will be returned
    - To edit user information or roles, click on user name
    - Delete user and add new user links are self-explanatory
- Available roles are selected via check boxes (not mutually exclusive), and authorization levels are as follows:
    - Admin – can perform all actions within the system
    - Clinical – has access to all functionality for patient, clinical samples, and dissection/extraction, including the ability to see MRN#. Can view sequencing libraries, and sequencing run information.
    - Clin_admin – same access as 'clinical' role, except can also modify drop-down lists for all categories of samples, and for H&E slide, pathology report
    - Researcher – has access to all functionality for dissected/extracted samples, sequencing libraries, flow cells and sequencing. Can view clinical sample information but cannot view MRN#.
    - Lab_admin – same access as 'researcher' role, except can also modify drop-down lists for sequencing libraries and sequencing runs.
    - Alignment – has access to all functionality for sequencing libraries, flow cells, sequencing and can modify alignment references and sequencing machines tables. Read access to other areas of the system, but cannot view MRN#.
    - Orders – can place orders for items.

Managing researchers

- Select Admin;Researchers from the top navigation bar
  List of researchers will be returned
    - Edit, Delete, and Add new researcher links are self-explanatory
- Note that researchers may be made 'inactive' if you wish to keep them as valid owners when editing an existing record (eg sequencing library), but not allow them to be a valid owner for a new record
